# Macrophage Dysfunction Impairs Resolution of Inflammation in the Wounds of Diabetic Mice

**DOI:** 10.1371/journal.pone.0009539

**Published:** 2010-03-04

**Authors:** Savita Khanna, Sabyasachi Biswas, Yingli Shang, Eric Collard, Ali Azad, Courtney Kauh, Vineet Bhasker, Gayle M. Gordillo, Chandan K. Sen, Sashwati Roy

**Affiliations:** Comprehensive Wound Center, Department of Surgery, Davis Heart and Lung Research Institute, The Ohio State University Medical Center, Columbus, Ohio, United States of America; Johns Hopkins School of Medicine, United States of America

## Abstract

**Background:**

Chronic inflammation is a characteristic feature of diabetic cutaneous wounds. We sought to delineate novel mechanisms involved in the impairment of resolution of inflammation in diabetic cutaneous wounds. At the wound-site, efficient dead cell clearance (efferocytosis) is a pre-requisite for the timely resolution of inflammation and successful healing.

**Methodology/Principal Findings:**

Macrophages isolated from wounds of diabetic mice showed significant impairment in efferocytosis. Impaired efferocytosis was associated with significantly higher burden of apoptotic cells in wound tissue as well as higher expression of pro-inflammatory and lower expression of anti-inflammatory cytokines. Observations related to apoptotic cell load at the wound site in mice were validated in the wound tissue of diabetic and non-diabetic patients. Forced Fas ligand driven elevation of apoptotic cell burden at the wound site augmented pro-inflammatory and attenuated anti-inflammatory cytokine response. Furthermore, successful efferocytosis switched wound macrophages from pro-inflammatory to an anti-inflammatory mode.

**Conclusions/Significance:**

Taken together, this study presents first evidence demonstrating that diabetic wounds suffer from dysfunctional macrophage efferocytosis resulting in increased apoptotic cell burden at the wound site. This burden, in turn, prolongs the inflammatory phase and complicates wound healing.

## Introduction

The Centers for Disease Control and Prevention (CDC) report that diabetes affects nearly 21 million Americans i.e., ∼7% of the U.S. population. Impairment of cutaneous wound healing is a debilitating complication commonly encountered during diabetes mellitus. Foot ulcers represent the most prevalent diabetic wounds and frequently lead to limb amputations. The incidence of diabetic foot lesions has been reported to be similar in type 1 vs type 2 diabetic patients [1]. In human diabetic ulcers, multiple deviations from normal healing have been identified (reviewed in [2]. Diabetic ulcers are characterized by a chronic inflammatory state primarily manifested by imbalances in pro- and anti-inflammatory cytokines [3]. Transient self-resolving inflammation is essential for successful wound healing. Wound inflammation is driven by a variety of mediators that are tightly controlled in space and time [4], [5]. Wound-site macrophages represent a key player that drive wound inflammation. Diabetes is known to compromise macrophage function including phagocytosis activity [6], [7]. Diabetic macrophages produce high levels of pro-inflammatory cytokines [8], [9].The causative factors underlying the chronic inflammatory state of diabetic wounds remain to be characterized.

During the early inflammatory phase, a large number of polymorphonuclear neutrophil (PMN nearly 50% of all cells at the wound site) are recruited to the wound site [10]. Following completion of their tasks, PMN must be eliminated in order to initiate the next stage of wound healing. Non-resolving persistent inflammation may derail the healing cascade resulting in chronic wounds. In the course of adult cutaneous wound healing, the granulation tissue decreases in cellularity and evolve into a scar [11]. Rapid increase in cell infiltration during tissue reconstruction is balanced by apoptosis. Apoptosis allows for the elimination of cells that are no longer required at the injury site or cells that are too damaged to facilitate the healing process. While mechanisms of apoptosis have been intensely studied, the specific mechanisms of disposal or clearance of apoptotic cells from the wound site remain poorly understood [12].

Phagocytosis of apoptotic cells has distinctive morphologic features and unique downstream consequences. deCathelineau and Henson [13] and Gardai et al [14] coined the term efferocytosis [15]. Efferocytosis refers to phagocytosis of apoptotic cells, an essential feature of immune responses and critical for the resolution of inflammation. This final removal step in the cell-death program plays a critical role in protecting tissues from exposure to the toxic contents of dying cells and also serves to prevent further tissue damage by stimulating production of anti-inflammatory cytokines and chemokines [16], [17]. Inappropriate clearance of cell corpses may lead to autoimmune diseases and chronic inflammation [18]. Adequate removal of apoptotic PMNs by macrophages from the wound site is a pre-requisite for the restoration of normal tissue function resolving inflammation. In the clinical treatment of chronic wounds, debridement is commonly practiced and is aimed at the removal of dead, damaged, or infected tissue to improve the healing potential of the remaining healthy tissue. We posit that at the wound-site successful debridement at the cellular level is a pre-requisite to the resolution of inflammation and successful healing. The objective of this study was to test this novel hypothesis and to delineate mechanisms that are involved in the impairment of resolution of inflammation in diabetic wounds. This reports presents first evidence collected from functionally active macrophages harvested from diabetic wounds.

## Results

**Figure 1:**
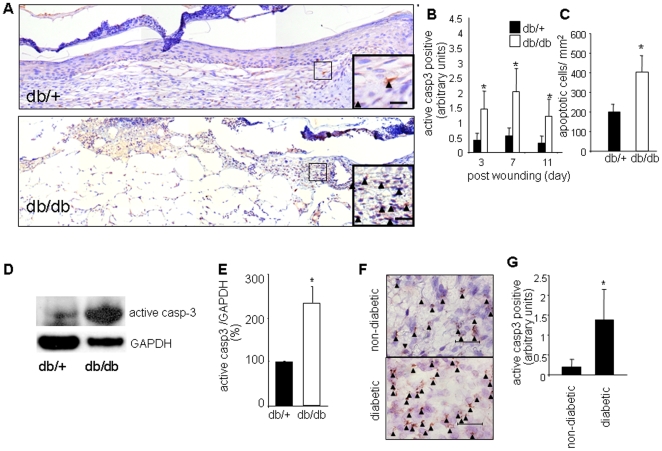
Increased number of apoptotic cells in wounds of diabetic mice and humans. **A**, Representative mosaic images from day 3 wounds of diabetic (db/db) or non-diabetic (db/+) mice stained with active caspase 3 (brown). Counterstaining was performed using hematoxylin (blue). The mosaic images of whole wounds were collected under 20× magnification guided by MosaiX software (Zeiss) and a motorized stage. Each mosaic image was generated by combining 12–14 images. *Inset:* higher magnification image of the boxed area marked in the mosaic image. scale bar (*inset*)  = 10 µm; **B–C**, quantification of active caspase 3 (**B**) or TUNEL positive cells (**C**). Data are shown as mean ± SD (n = 3); *, *p*<0.05 *versus* control non diabetic (db/+) mice; **D**. A representative Western blot image of active caspase-3 (casp-3) and GAPDH (housekeeping) in day3 wound tissue extracts of diabetic (db/db) or non-diabetic (db/+) mice. **E**. Densitometry data of blot shown in panel D. Data shown are mean ± SD (n = 3). *, p<0.05 compared to db/+ mice; **F–G**, wound biopsies were obtained from non-diabetic or diabetic patients presented at the wound clinic. Specimens (3 mm punch) were obtained from the edge of wounds immunostained using active caspase 3 (brown) antibody as a marker of apoptotic cells. Counterstaining was performed using hematoxylin (blue); **F**. microscopic images, arrows indicate positive cells. Scale bar  = 50 µm; **G**, quantification of active caspase 3 positive areas shown in F. Data shown are mean ± SD (n = 3); * *p*< 0.05 versus non diabetic leg wounds.

**Figure 2:**
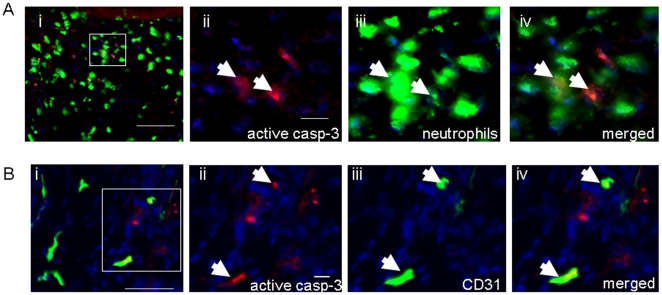
Identification of apoptotic neutrophils and endothelial cells in diabetic wounds. Representative immunostained section of: **A**, day 3 wound showing neutrophils (green) and active casp-3 (red) staining; and **B**, day 7 wound showing endothelial cells (CD31, green) and active casp-3 (red) staining. Nuclear counterstaining was performed using DAPI (blue). *i*, Low power (20x) images, Scale bar  = 50 µm.; *ii-iv*, high powered images of the boxed area in *i* showing active casp-3 (red) and DAPI (blue) (ii) anti-neutrophil or anti-CD31 (green) and DAPI (blue) (iii); and merged images of anti-neutrophil/anti-CD31 and active casp-3. Casp3 positive neutrophils/endothelial cells are shown with white arrows. Scale bar (**A**ii-iv and **B**ii-iv)  = 10 µm.

Mice homozygous (BKS.Cg-m +/+ Leprdb/J or db/db) for spontaneous mutation of the leptin receptor (Lepr^db^) become identifiably obese around 3 to 4 weeks of age. Elevation of blood sugar was evident within 4 to 8 weeks after birth. Excisional (6 mm) wounds created on the back of these mice showed severe impairment in closure when compared to their corresponding control (heterozygous db/+) mice (data not shown). Using histological approaches, we observed that the wounds of diabetic mice contained higher number of apoptotic cells compared to the wound tissue of control db/+ mice (Figs. 1A–B). TUNEL staining and Western blot using active caspase-3 antibody demonstrated consistent outcomes (Fig. 1C–E). To test the clinical relevance of the above-said findings punch biopsies (3 mm) were collected from matched (patient characteristics, wound location and clinical condition) wounds of consented diabetic and non-diabetic patients (Table 1). Scoring of active caspase 3 positive cells demonstrated a significantly higher load of apoptotic cells in the wound tissue of individuals with diabetes (1F–G). In order to identify the types of cells in the diabetic wounds that were undergoing apoptosis, dual immunofluorescence studies were performed (Fig. 2). Wound tissue sections (d3 and d7) from diabetic animals were immunostained with active caspase-3 antibody to visualize apoptotic cells. The sections were then co-immunostained using either anti-neutrophil or anti-CD31 (endothelial cell marker) antibody Fig. 2A&B). The results clearly demonstrate that at day 3 majority of caspase-3 positive cells were neutrophils. Some endothelial cells were positive for caspase-3 on day 7 post-wounding. These data suggest PMNs, and endothelial cells at least in part, represent the apoptotic cells detected in the wounds.

**Table 1:** Demographic characteristic of patients and their wound size/age.

	Control	Diabetic
**Age**	48±5	45±11
**Gender**	M,F	M,F
**Race**	AA,C	AA,C
**Wound size (mm^3^)**	2.55-82.11	1.26-66.0
**HbA1c**	ND	4.7-6.0
**Wound age**	>30 d	>30 d

AA, African American; C, Caucacians; M, male; F, female; ND, not determined;

To test whether increased oxidative stress in diabetic mice contributes to increased number of apoptotic cells in wounds, the diabetic mice were supplemented (gavaged) daily with N-acetyl cysteine (NAC). NAC is a well known antioxidant effective in decreasing oxidative stress in diabetic mice at the abovementioned dose [19], [20]. After three weeks of supplementation, plasma lipid peroxidation levels were measured as marker for oxidative stress. Data presented in Fig. S1A demonstrate that diabetic mice, compared to the matched non-diabetic mice, show significantly high levels of lipid peroxidation. NAC supplementation for three weeks significantly decreased plasma lipid peroxidation (Fig. S1A). 4-Hydroxy-2-nonenal (HNE) is a major product of endogenous lipid peroxidation, which is found as a footprint in the aftermath of oxidative stress [21]. Anti-HNE staining demonstrated that similar to plasma, wound tissue of db/db mice exhibit higher levels of oxidative stress which is ameliorated following NAC supplementation (Fig. S1B). Despite attenuated oxidative stress following NAC supplementation, no change in apoptotic cell count in wounds was observed in the diabetic mice (Fig. S1C). These observations argue against the involvement of oxidative stress in determining the apoptotic cell load in the wound tissue of diabetic mice.

**Figure 3:**
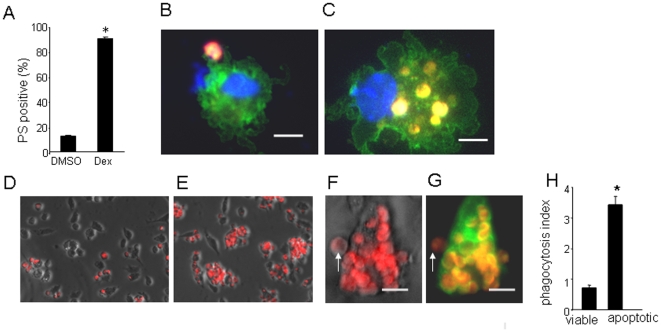
Dead cell clearance by wound-site macrophages. For dead cell clearance assay, wound macrophages were co-cultured with cell-tracker labeled (red) thymocytes. **A**, Thymocyte apopotosis detected using Annexin V (FITC conjugated). Annexin V binds to externalized phosphatidyl serine (PS), a characteristics of apoptotic cells. Such treatment resulted in over 90% cells becoming phosphatidylserine (PS) positive. Data are mean ± SD; p<0.05 (n = 4). **B**, F4/80-FITC (green) and DAPI (blue, nuclear) stained wound macrophage establishing link with an apoptotic thymocyte (red); **C**, wound macrophage (F4/80-FITC and DAPI stained) engulfed a number of red apoptotic thymocytes; **D**, co-cultures of control (untreated, *viable*) thymocytes (red) with wound macrophage (DIC image) followed by wash; **E**, co-cultures of apoptotic thymocytes (red) with wound macrophages (differential image contrast, DIC image) followed by wash; **F–G**, Representative high magnification image of a macrophage in DIC (**F**) or stained with F4/80 FITC (green, **G**) showing engulfed and adhered (white arrows) apoptotic thymocytes (red). **H**, scoring of thymocytes engulfed by macrophage. Data are presented as *phagocytic index* which is defined as total number of apoptotic cells engulfed by macrophages in a field of view divided by total number of macrophage presented in the view. This approach enables normalization of the data against macrophage number. Data presented as mean ± SD (n = 3). *, *p*<0.01 compared to macrophage co-cultured with control thymocytes.

**Figure 4:**
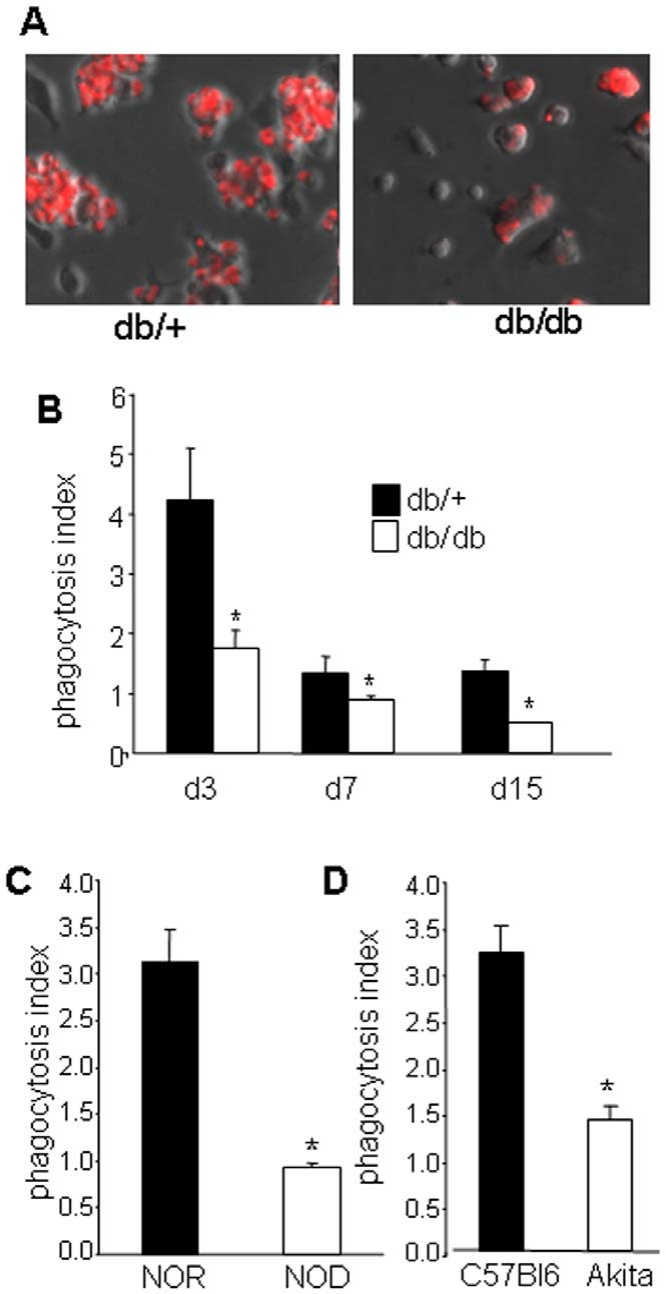
Dead cell clearance activity is impaired in wound-site macrophages harvested from diabetic mice. **A**, representative images of macrophage (phase contrast) from diabetic (db/db) and their matched control non diabetic (db/+) co-cultured with apoptotic thymocytes (red); **B–D**, quantification of dead cell clearance activity of wound macrophages from three different genetic backgrounds; **B**, phagocytic index of wound macrophages harvested 3, 7 or 15 day post implantation from db/+ (non-diabetic control) or db/db (type 2 diabetes); **C**, phagocytic index of wound macrophages harvested day 5 post implantation from NOR (control) or NOD (type 1 diabetes); and **D**, phagocytic index of wound macrophages harvested day 5 post implantation from Akita (Ins2Akita, type 1 diabetes) & C57Bl6 (non-diabetic controls). Data are mean ±SD (n = 3).*, *p*<0.05 versus control mice.

To evaluate apoptotic cell clearance activity, homogenous wound macrophage suspensions were derived from diabetic and non-diabetic (control) mice. Wound macrophages were isolated employing a polyvinyl alcohol (PVA)-sponge implantation approach. This isolation procedure resulted in macrophage cultures with >95% (95.9±1.7%) homogeneity. Wound-site macrophages were co-cultured with cell-tracker (red)-tagged cells that were either apoptotic or viable (Fig. 3). Thymocytes were made apoptotic by activating them with 5 µM dexamethasone for 12 h. Over 90% cells become phosphatidylserine (PS) positive (Fig. 3A). The co-culture resulted in clasping (Fig. 3B) and phagocytosis of the fluorescent-labeled apoptotic cells (Figs. 3C). Macrophages were phagocytotically-silent when co-cultured with viable cells (Fig. 3D). The difference in observation between Figs. 3D&E were objectively tested using scores (Fig. 3H). High powered DIC or fluorescence images were utilized to discriminate between adherent and engulfed apoptotic cell (Fig. 3F&G). Significantly impaired apoptotic cell clearance or efferocytosis activity of wound macrophages isolated from diabetic mice was noted (Fig. 4). We sought to test whether the impairment in apoptotic cell clearance activity of wound macrophages was limited to the db/db model or macrophages from other diabetic mice models show comparable results. To address this issue, we used other genetic models of type 1 diabetes. The NOD/LtJ mice are susceptible to spontaneous development of autoimmune (type 1) insulin dependent diabetes mellitus (IDDM) [22]. Similarly, B6-Ins2Akita model of spontaneous type 1 diabetes is a relatively new model of non-obese insulin-dependent diabetes [22]. Consistent with results from the db/db mice, wound macrophages derived from NOD as well as from Akita mice showed increased number of apoptotic cells in wound tissue (data not shown) as well as clear impairments in dead cell clearance activity (Figs. 4C&D). Results addressing the time-course demonstrate that wound macrophages harvested form non-diabetic mice during the early inflammatory phase (day 3 post-wounding) possess the highest apoptotic cell clearance activity (Fig. 4B). This activity is attenuated during the intermediary (day 7) or late (day 15) phases of healing (Fig. 4B). Compared to controls, the phagocytic activity was markedly impaired in macrophages from diabetic mice at all time points examined (Fig. 4B).

**Figure 5:**
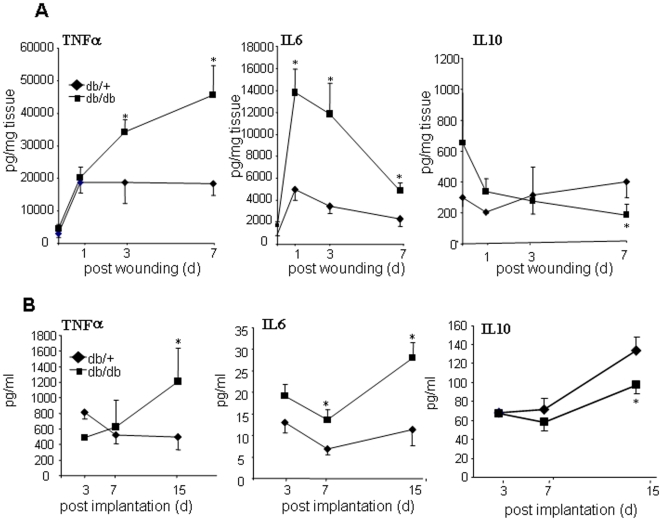
Increased pro-inflammatory cytokine levels in diabetic wounds and in wound-site macrophages. **A**, Cytokine levels in excisional wound tissue collected on days 1, 3 and 7 post-wounding were measured using ELISA. Data are presented as *pg* cytokine levels per *mg* of wet tissue. Mean ± SD (n = 5).*, *p*<0.05 db/db *versus* db/+; **B**, PVA sponges were harvested on days 3, 7 or 15 after implantation and macrophages were isolated. Macrophages (1×10^6^) were seeded in 6-well plates. Cytokine levels in culture media was measured 24 h post-seeding using ELISA. Mean ± SD (n = 4). *, *p*<0.05 db/db *versus* db/+.

Transient inflammation is an integral component of the successful healing process [23]. While inflammation-derived mechanisms support key healing processes such as debris-removal and angiogenesis, it is necessary that the inflammation be resolved in a timely manner to allow the remainder of the healing cascade to follow [23]. To address mechanisms implicated in the resolution of wound inflammation, wound tissue was harvested from diabetic mice and their corresponding controls on days 1, 3, and 7 day post-wounding. Compared to corresponding control mice, diabetic mice showed increased levels of the pro-inflammatory cytokines TNF-α and IL-6. Of related interest, IL-10, an anti-inflammatory cytokine, was significantly lower in the diabetic wound tissue (Fig. 5A). This line of evidence demonstrates an imbalance between pro-inflammatory and anti-inflammatory cytokines in the diabetic wound antagonizing timely resolution of inflammation. Macrophages represent a major source of cytokines in the wound. Thus, cytokine production by wound macrophages isolated from diabetic and control mice was examined. Wound macrophages from db/db and db/+ control animals were isolated and cultured overnight. Next, the pro-inflammatory cytokines TNFα, IL-6 were assayed from the culture media. Macrophages from diabetic mice produced higher levels of the pro-inflammatory cytokines TNF-α & IL-6 and lower anti-inflammatory cytokine IL-10 compared to non-diabetic controls (Fig. 5B). Taken together, the results from wound tissue (Fig. 5A) as well as isolated wound-site macrophages (Fig. 5B) demonstrate that the cytokine expression pattern in the diabetic wound resist resolution of inflammation leading to a prolonged inflammatory phase.

**Figure 6:**
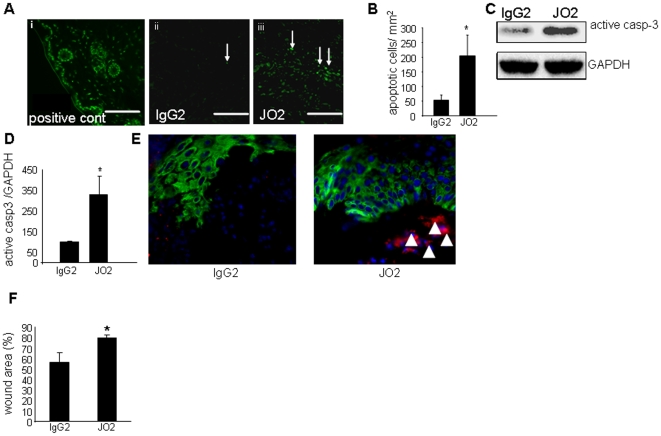
Topical application of Fas-activating anti-CD95 JO2 to the wound-site increased apoptotic cell count while not inducing apoptosis in keratinocytes. **A**, visualization of TUNEL stained apoptotic cells in day 5 wound tissue treated with anti-murine CD95 (clone:JO2, 2 µg/wound) or vehicle containing isotype control (IgG2). Positive control (wound tissue treated with proteinase K and nuclease) showing TUNEL positive apoptotic cells with green nuclei stain; **B**, scoring of apoptotic cells in wound tissue sections stained with TUNEL. *, *p*<0.05 compared to the paired vehicle-treated wounds; **C**, a representative Western blot image of active caspase-3 (casp-3) and GAPDH (housekeeping) in tissue extracts from IgG2 or JO2 treated d3 wounds; **D**, densitometric data of blot shown in panel C. Data shown are mean ± SD (n = 3). *, p<0.05 compared to IgG2 treated wounds; **E**, JO2 treatment did not induce keratinocyte apoptosis. Keratin-14 (green), active caspase-3 (red) and DAPI (blue) stained migrating epithelial tip in placebo (left) or JO2 treated (right) wounds. Scale bar  = 20 µm. Active caspase-3 staining was observed in the granulation tissue but not in the hyper-proliferative epithelium or epithelial tip following JO2 treatment; **F**, wound area as percentage of initial wound determined on the day 3 after wounding. Data are shown as mean ± SD (n = 4).*, p<0.05 versus corresponding control IgG2 treated wound.

**Figure 7:**
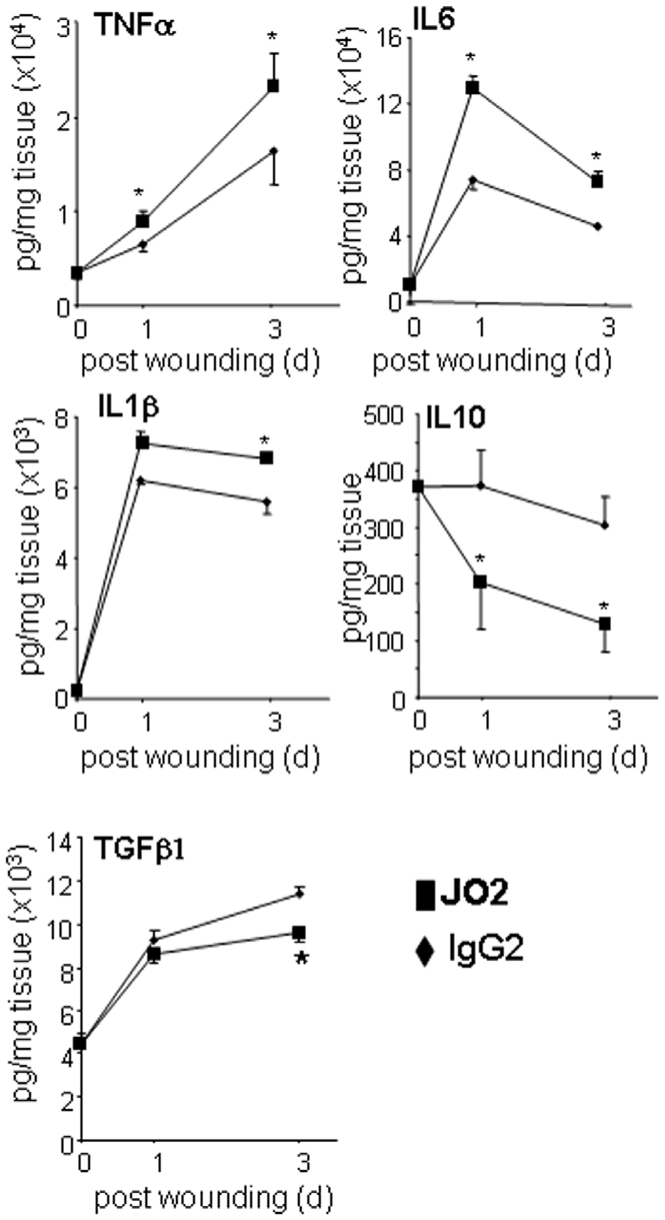
Increasing dead cell burden in wounds resulted in increased pro-inflammatory cytokine levels. Wound tissue treated with anti-CD95 JO2 or control IgG2 were harvested on days 0, 1 and 3 post-wounding. Cytokine levels from paired (control and treated) wound tissue were measured using ELISA on the indicated days post-wounding. Significant increase in pro-inflammatory cytokines (TNFα, IL-6, IL1β) and decrease in levels of anti-inflammatory cytokines IL-10 and TGFβ1 was noted in wounds that had increased apoptotic cell load. Data (mean ±SD, n = 5) are presented as *pg* cytokine per *mg* wound tissue. *, *p*<0.05 compared to IgG treated control side.

In the next phase of the study we sought to address whether there is a link between the observed higher burden of apoptotic cells in the diabetic wound and impaired resolution of wound inflammation and closure. To increase apoptotic cell burden at the wound site with minimal perturbation of other aspects of wound biology, JO2 (anti-CD95) or its isotype control (IgG2) antibody was applied topically to wounds once per day during the early inflammatory phase (0–4 d post wounding). This approach led to a significantly higher load of apoptotic cells at the wound site (Fig. 6A–D). The Fas antigen is a death-domain containing cell surface protein that is present in many cell types [24]. Our goal, in using this approach, was to increase apoptotic cell burden caused by fas-mediated killing of neutrophils. However, we recognize that there is a possibility that other major cell types *e.g.* keratinocytes and macrophage present at the wound tissue may be killed by JO2. Thus, we chose to experimentally address this potential complication. Results showed that growing keratinocyte tip cells were not affected by anti-CD95 JO2 treatment (Fig. 6E). Wound macrophages were also not affected by anti-CD95 JO2 treatment (data not shown). These findings are consistent with published reports showing that these two cell types both are resistant to CD95-mediated apoptosis [25], [26]. Of outstanding interest was the observation that increasing apoptotic-cell count at the wound site caused significant increase in pro-inflammatory cytokine (TNFα, IL-1β, and IL-6) expression and decreased anti-inflammatory cytokine (IL-10 and TGFβ1) levels at the wound site on days 1 & 3 post-wounding (Fig. 7). Such an augmented inflammatory response in JO2 treated wounds was associated with impaired closure as manifested by expanded wound area (Fig. 6F).

**Figure 8:**
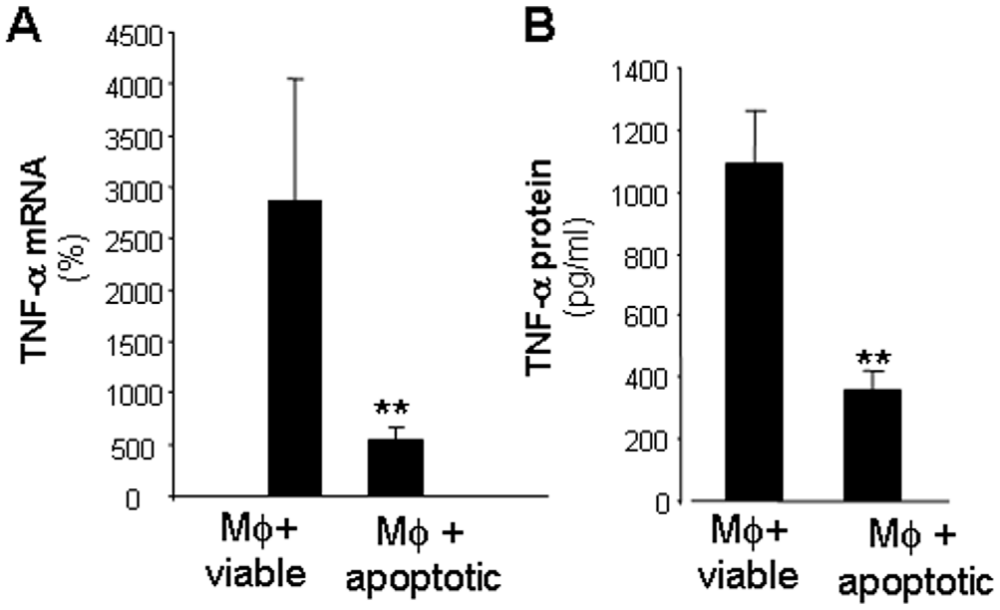
Efferocytosis of apoptotic cells by wound macrophages resulted in suppression of pro-inflammatory TNFα gene and protein expression. Following apoptotic cell clearance assay the non-phagocytosed thymocytes were removed by washing and cells were challenged with LPS (1 µg/ml) and IFNγ (10 ng/ml) for 4 h (gene expression) or 16 h (protein expression). TNFα gene (**A**) and protein (**B**) expression were measured using real-time PCR and ELISA, respectively. mRNA expression data are presented as % change compared to LPS+IFNγ non-activated control samples. Protein data is expressed as concentration of TNF-α secreted in culture media. Data are mean ±SD (n = 4); **, *p*<0.01 compared to macrophage cultured with viable cells.

Finally, we tested the hypothesis that successful efferocytosis “switches” the wound macrophages from a pro-inflammatory mode to an anti-inflammatory mode. Following effecrocytosis wound macrophages were activated with LPS and IFN-γ to induce expression of TNF-α, as a marker of pro-inflammatory mediator. A significant suppression of inducible TNFα gene and protein expression was observed in post-phagocytosis macrophages (co-cultured with apoptotic cells) compared to macrophages that did not phagocytose (cultured with viable cells) (Fig. 8). These observations indicate that successful phagocytosis suppresses pro-inflammatory gene expression in macrophages leading to the concept that impaired efferocytosis in the diabetic wound may be responsible for defective resolution of inflammation.

## Discussion

Diabetes is known to be associated with impaired phagocytic function of macrophages [27], [28], [29], [30], [31], [32]. Findings of this study collectively present maiden evidence supporting that increased count of apoptotic cells in cutaneous wounds of diabetic mice and humans is associated with compromised dead cell clearance activity of wound macrophages. The major conclusions of this study are that: *i)* diabetic wounds have increased apoptotic cells load which is in part due to impaired apoptotic clearance activity of the macrophages at the diabetic wound site. This conclusion is based on the observations that diabetic wounds in mouse and human have increased apoptotic cell count primarily contributed by apoptotic PMNs, and that macrophages isolated from diabetic wounds are impaired in their activity to phagocytose apoptotic cells; *ii)* increase in apoptotic cell burden in diabetic wounds augments inflammatory response in wounds. This conclusion is based on the observation that experimental elevation of apoptotic cell load at the wound site resulted in increased inflammatory response; and *iii)* impaired dead cell clearance activity in diabetic wound macrophages compromises resolution of inflammation in diabetic wounds. This is supported by the observation that successful clearance of apoptotic cell by wound macrophages attenuates the expression of inflammatory cytokines and that diabetic wound macrophages, impaired in their ability to phagocytose, produce elevated levels of pro-inflammatory cytokines.

Elevated apoptotic cell count is a known feature of the diabetic wound [33]. Factors other than impaired phagocytosis by macrophages, as proposed in this study, that may contribute to elevated apoptotic cell count in the diabetic wound include acceleration of apoptosis caused by advanced glycation end products (AGEs), activation of protein kinase C (PKC) and increased oxidative stress [34]. In support of glycation being involved in accelerating apoptosis of neutrophils in the peripheral blood of patients with T2DM a tight association between the rates of apoptosis with elevated HbA1c has been reported [35]. Implication of oxidative stress in accelerating apoptosis in diabetics have been proposed [36] but is not supported by observations of the current study demonstrating the effectiveness of NAC as an antioxidant but no effect of such intervention on apoptotic cell burden at the wound site.

Other than accelerated apoptosis, increased apoptotic cells load at the wound site may be contributed by inefficient clearance of apoptotic cells from wounds by macrophages. Peritoneal macrophages from non-obese diabetic (NOD) mice are known to engulf apoptotic cells less efficiently than those from non-diabetic mice [30], [32]. Our observation that wound derived macrophages from three different genetic models of diabetes suffer from impaired efferocytosis function is consistent with such reports. From a metabolic and related mechanistic stand-point, NOD and db/db diabetic mice have several fundamental differences [22], [30], [37]. NOD mice develop spontaneous autoimmune destruction of β-cells at approximately 5 month of age. This model has a number of similarities with features of human type 1 diabetes [22]. In terms of early onset and an autosomal dominant mode of inheritance and primary dysfunction of the β cells, Akita mice resemble the condition of human maturity-onset diabetes of the young [38]. Db/db mouse is an established model of deficient wound healing associated with human type 2 diabetes mellitus (T2DM) [39], [40]. The genetic basis for this inbred mouse model is a single-gene autosomal recessive defect in leptin receptor which produces leptin resistance and results in hyperphagia, obesity and the subsequent symptoms of insulin resistance, insufficient insulin secretion, hyperglycemia and elevated HbA1c (9.1±2.1) levels [22], [41], [42], [43]. One common feature of three above-described models is that they all suffer from hyperglycemia. Hyperglycemia associated advanced glycated end products (AGEs) have been shown to directly suppress phagocytosis activity of macrophages [31].

Inflammation is tightly regulated by the following two types of signals: *i)* initiate & maintain inflammation; and *ii)* resolve inflammation [23]. An imbalance between the two signals, in favor of the former, results in chronic inflammation and derails the healing cascade. Pro-inflammatory cytokines IL-1α, IL-1β, IL-6 and TNF-α are prominently up-regulated during the repair process [44]. IL-10 is recognized as a major suppressor of the inflammatory response [45]. An important role of this anti-inflammatory cytokine in attenuating the expression of pro-inflammatory cytokines in fetal wounds resulting in minimized matrix deposition and scar-free healing has been demonstrated [46]. Increased levels of the pro-inflammatory cytokines TNF-α and IL-6 and a decreased level of IL-10, an anti-inflammatory cytokine were observed in diabetic wound tissue compared to non-diabetic healing wound. A modest yet significant decrease in IL-10 levels in db/db mice wound compared to non-diabetic control suggests that IL-10 alone is not sufficient in suppressing the augmented inflammatory response in the wounds of these mice. Interestingly, increased expression of IL-10 is known to be associated with impaired healing in humans chronic venous insufficiency ulcers [47] that are known to have persistent inflammation. The specific significance of IL-10 in regulating diabetic wound inflammation remains to be characterized. Persistent (day 13 post wounding) expression of the inflammatory cytokines IL-1α and TNF-α was observed in an excisional wound healing model in diabetic (db/db) mice [48]. Lowering of the functionally available levels of the pro-inflammatory cytokine TNF-α using anti-TNF-α therapy directed at managing activated macrophages restore diabetic wound healing in ob/ob mice [9]. These lines of evidence suggest that the perturbation of a delicate balance between pro-inflammatory and anti-inflammatory cytokines in the diabetic wound predisposes the wound to impaired resolution of inflammation [3], [48]. Macrophages represent a major source of the cytokines in wounds [44]. The kinetics of cytokine production by wound macrophages was not concurrent with the dynamics of tissue wound cytokines. However, the observation that macrophages derived from diabetic wounds produces increased levels of pro-inflammatory cytokines compared to the non-diabetic control macrophages support that the augmented pro-inflammatory state of diabetic wound tissue is at least in part due to increased pro-inflammatory phenotype of the diabetic wound macrophages. This contention is supported by studies using macrophage depleted mice where a key role of macrophages in wound cytokine/growth factor dynamics has been demonstrated [23], [49], [50]. TNF-α expressing macrophages in diabetic animals have been also shown to be are primarily responsible for the impairment of wound healing in this genetic model [9] The macrophages isolated from CD18 null mice exhibit impaired phagocytic clearance of PMNs, impaired wound closure and a marked reduction of TGF-β1, an anti-inflammatory growth factor released by macrophages [51]. These two studies further support the contention that the pro-inflammatory state of wound macrophage plays a major role in compromising the resolution of inflammation in diabetic wounds.

The Fas/Fas ligand pathway has been implicated as an important cellular pathway mediating apoptosis in diverse cell types [52]. Neutrophils are specifically highly susceptible to rapid apoptosis *in vitro* after stimulation with activating anti-Fas IgM (mAb CH-11) [53]. Topical treatment of anti-CD95 JO2 treatment does not induce apoptosis in proliferating keratinocytes or macrophages. We utilized this opportunity to set up a Fas-directed approach to increase the apoptotic cell burden at the wound site. The approach reported in this study served as an effective tool to query the significance of apoptotic cell burden on wound biology. Higher apoptotic cell burden at the wound-site resulted in larger open wound indicating a slower rate of closure. Contraction, epithelialization and granulation tissue formation represent the major processes that contribute to the overall wound healing/closure of full thickness dermal wounds. No effect on wound epithelialization yet a slower rate of closure suggests that contraction and/or granulation tissue formation was likely affected under these conditions. Increased apoptotic cells in wounds also resulted in elevated the levels of pro-inflammatory cytokines in wounds supporting the notion that increased apoptotic cell burden at the wound-site results in augmented inflammatory response. This is consistent with previous observation in non-wound studies demonstrating that inappropriate clearance of apoptotic cell corpses lead to chronic inflammation [18]. Both apoptosis as well as the efficient clearance of apoptotic cells are important determinants of the resolution of inflammation *in vivo* [54], [55], [56]. Phagocytic removal of apoptotic cells by macrophages is a pre-requisite for the restoration of normal tissue function resolving inflammation [56], [57], [58], [59]. Engulfment of apoptotic cells by macrophages results in potent anti-inflammatory and immunosuppressive effects caused by production of anti-inflammatory cytokines such as TGF-β1, IL-10 & IL-4 and suppressed release of pro-inflammatory mediators including TNF-α, IL-6 by activated macrophages [56], [60], [61], [62].

Macrophages are dynamic and heterogeneous cells. Since the introduction of the concept of alternative activation of macrophages in 1992 [63], these cells have been broadly assigned to two broad groups: (i) classically activated or type I macrophages (M1) which are pro-inflammatory effectors, and (ii) alternatively activated or type II macrophages (M2) that possess anti-inflammatory properties [64]. In response to cues from the microenvironment, pro-inflammatory activated M1 macrophages may switch to M2 [65]. Results of this work support that efferocytosis may be one of such cues that drives the switching of macrophages towards an anti-inflammatory state [60].

While efferocytosis orchestrates successful resolution of inflammation, this process is also regulated in an autocrine manner by anti- as well as pro-inflammatory mediators such as TNFα [66]. Once inflammation is resolved, the phenotype of resolution-phase macrophages has been shown to be altered primarily via cAMP dependent mechanisms [67]. For the first time, we present functional results from viable macrophages isolated from the wound-site *in vivo* to directly demonstrate that successful efferocytosis of apoptotic cells results in suppression of a major pro-inflammatory mediator *i.e,* TNFα. Understanding the mechanisms of resolution of inflammation by wound macrophages as well as of the post resolution phenotype of macrophages require further investigation.

In sum, this study provides first evidence that macrophages from diabetic wounds suffer from impaired in dead cell clearance activity as one of the key factors resulting in increased apoptotic cell burden at the wound site. This burden, in turn, prolongs the inflammatory phase and complicates the healing process and compromises resolution of inflammation. Correction of impaired efferocytosis in diabetic wounds and strategies to intercept the adverse effects of impaired efferocytosis emerge as novel targets for the management of chronic inflammation commonly noted in diabetic wounds.

## Materials and Methods

### Ethics Statement

#### Vertebrate animals

All animal studies have been approved by Ohio State University's Institutional Animal Care and Use Committee (IACUC).

#### Human subjects

All human studies were approved by the Ohio State University's Institutional Review Board (IRB).

### Secondary-Intention Excisional Cutaneous Wound Model

Male (8–12 week aged) mice were used for this study. For wounding, mice were anesthetized with isoflurane inhalation. Two 6 mm full-thickness (skin and panniculus carnosus) excisional wounds were placed on the dorsal skin (shaved and cleaned using betadine), equidistant from the midline and adjacent to the four limbs. The wound were left to heal by secondary intention [68], [69], [70].

#### Determination of wound area

Imaging of wounds was performed using a digital camera (Canon PowerShot G6). The wound area was determined using WoundMatrix™ software as described previously [68], [69], [70]. All animal studies have been approved by Ohio State University's Institutional Animal Care and Use Committee (IACUC).

### Polyvinyl Alcohol (PVA) Sponges Implantation

Circular (8 mm) sterile PVA sponges were implanted subcutaneously on the back of the mice, a location matched for the site of excisional wounds [71]. In brief, following induction of anesthesia by isofluorane inhalation, dorsal midline was shaved and cleaned with betadine. Two midline 1 cm incisions were made with a scalpel. Small subcutaneous pockets were created by blunt dissection, two pockets per animal. Two PVA sponges were inserted to each pocket. Incisions were closed with skin staples (9 mm) or suture (3-0 Surgiline™). Animals were then returned to clean cages for the monitoring of recovery. The animals were euthanized by CO_2_ inhalation for final harvest of the PVA sponges.

### Isolation of Wound Macrophages from PVA Sponges

Subcutaneously implanted PVA sponges were harvested on a designated day and a single wound cell suspension was generated from sponges by repeated compression. The cell suspension was filtered through a 70 µm nylon cell strainer (Falcon) to remove all the sponge debris. For macrophage isolations, magnetic cell sorting was carried out using mouse anti-CD11b tagged microbeads (Miltenyi Biotec, Auburn, CA). This procedure yields a purified (>95%) population of wound macrophage as determined by F4/80 staining. Subcutaneously implanted polyvinyl alcohol (PVA) sponges are extensively used as model for wound-healing studies especially those addressing inflammation [71], [72]. The model is best suited for acute studies because on a longer term, after about 4 weeks of implantation, it is known to elicit foreign body response resulting in giant cell accumulation and fibrosis. In shorter term studies, the approach represents a reproducible and biologically valid model for the study of acute healing responses [72]. No major differences were noted in the cell characteristics and activity of PVA sponge derived cells and closed incisional wound derived cells [72]. When compared to excisional wounds, some differences that are primarily attributed to low-grade bacterial contamination of open wounds have been reported [73]. In our excisional wound studies, we routinely check wounds for bacterial contaminations using procedures described previously [69]. As reported earlier although low grade contamination is indeed observed in superficial tissues, deep tissue biopsies have not shown any bacterial contamination [69] suggesting that excisional wound deep-tissue macrophages are similar to macrophages derived from the described PVA sponge model.

### Apoptotic Cell Clearance Assay

For the assay, wound macrophages were seeded in 8-well chambered slides. Apoptotic (5 µM dexamethasone treated for 12 h; yield >90% PS positive thymocytes, Fig. 3A) thymocytes were added to each chamber in a (1∶10) macrophage:thymocyte ratio. Prior to co-culture with macrophages, thymocytes were labeled with a fluorescence cell-tracker reagent (CellTracker™ Orange CMTMR, Molecular Probes). Thymocytes have been largely used and are well accepted for efferocytosis studies performed using cultured macrophage *ex vivo*. Moreover, upon induction of apoptosis, both PMN and thymocytes are known to externalize phosphatidyl serine (PS), one of the key mechanisms of apoptotic cell recognition by macrophages [74], [75]. Phagocytosis assay was performed for 1 h at 37°C. In co-culture studies, shorter incubation times (10–15 min) were used for adherence assay while longer (45–60 min) co-culture period were utilized for the phagocytosis assays [51]. Macrophages were then extensively washed to remove non-phagocytosed cells. Cells were fixed with 4% paraformaldehyde and stained using F4/80-FITC. Imaging was performed using a fluorescence microscope (thymocytes, red; macrophage green or phase contrast). Quantitation of phagocytosed thymocytes by each macrophage was performed using Axiovision software (Zeiss) by counting 50–100 macrophages from each well. Data are expressed as “phagocytic index”. This index is defined as the total number of apoptotic cells engulfed per macrophage present in the field of view [62]. This approach enables normalization of the data against macrophage number.

### Human Subjects and Sample Collection

Subjects participating in the study were chronic wound patients seen at our Comprehensive Wound Center outpatient clinics that have been either clinically diagnosed type 2 diabetes (n = 3) or no diagnosis of diabetes (non-diabetic, control; n = 3). The demographic characteristics of patients and wound related information are listed in Table 1. Protocols were approved by the Ohio State University's Institutional Review Board. Declaration of Helsinki protocols were followed and patients gave their written, informed consent. Wound (at the wound perimeter) biopsies (3 mm) were obtained from individual subjects, immediately embedded in O.C.T compound (Tissue-Tek®) and stored frozen in liquid N_2_ for histological analysis.

### Histology

Formalin-fixed paraffin-embedded or frozen wound specimens were sectioned. Frozen section (10 µm) or deparaffinized paraffin sections (4 µm) were immunostained as described earlier [69] using the anti-active caspase 3 (anti-active caspase 3, Abcam, Inc, Cambridge, MA) and rabbit anti-HNE (Alexis, AXXORA, LLC, san Diego, CA) antibody. The sections were subsequently stained using appropriate HRP or fluorochrome tagged secondary antibody and counterstaining were performed as described previously [69]. For the visualization of wound epithelialization, anti-keratin-14 (1∶500; Covance, Berkeley, CA) with appropriate fluorescence tagged secondary antibody was used. Counterstaining was performed with DAPI to visualize nuclei (Molecular probe, OR). Neutrophils and endothelial cells were visualized using anti-neutrophil (1∶100, Serotec, Raleigh, NC) and CD31 (1∶200, BD Pharmingen, San Diego, CA).

#### TUNEL staining

This assay enables the monitoring of apoptotic cells in tissue sections and was performed using a commercially available kit (DermaTACS, Trevigen Inc).

#### Image quantification

Between 3–5 high powered images were quantified for each data point from each animal. Quantification was performed employing a Image processing tool kit (Adobe Photoshop) software that utilizes a color subtractive process [76].

### Antioxidant Supplementation

Diabetic (db/db) mice were divided in two equal subgroups. The first group was supplemented (intragastric, daily once) with *N*-acetylcysteine (NAC) at a dose of 1 mg/g body weight [19], [20]. The control group of mice was supplemented with matched volume of the vehicle (saline).

### Plasma Lipid Peroxidation

As a marker of lipid peroxidation, plasma malondialdehyde (MDA) levels were detected by the thiobarbituric acid reactive substances (TBARS) method. The assay was performed using OXItek TBARS assay kit (ZeptoMetrix Corporation, Buffalo NY).

### Wound Tissue Cytokine Analysis

Wound edge tissue [70] was pulverized under liquid nitrogen followed by extraction of protein in a buffer compatible with ELISA as described [77]. The cytokine levels in tissue extracts were measured using commercially available ELISA kits (R & D Systems).

### Wound Macrophage Cytokine Analysis

Macrophages were seeded in 6-well plates and cultured in RPMI 1640 medium containing 10% heat inactive bovine serum for 24 h under standard culture conditions. After 24 h, the media was collected and the cytokine levels in culture media were measured using commercially available ELISA kits (R & D Systems).

### Western Blot

Western blot was performed as described previously [78], [79], [80]. Primary antibody against active caspase-3 was obtained from Abcam.

### Statistics

*In vitro* data are reported as mean ± SD of at least three experiments. Comparisons among multiple groups were made by analysis of variance ANOVA. *p*<0.05 was considered statistically significant. For *in vivo* studies, data are reported as mean ± SD of at least 4–6 animals and 3 humans per group.

## Supporting Information

Figure S1 Antioxidant supplementation to diabetic mice attenuates oxidative stress but does not influence apoptotic cell count in wound tissue. Diabetic db/db mice were supplemented with N-acetyl-cysteine (NAC, 1 mg/g body weight, daily once) for three weeks. The control db/db group was supplemented with matched volume of saline. At the end of three weeks blood glucose, body weight and plasma MDA levels were measured. Two excisional (8 mm punch) wounds were placed on the back of mice. NAC supplementation continued throughout the healing period. A, Plasma lipid peroxidation (MDA levels) as a marker of oxidative stress was measured. Data are mean ± SD; n = 6. **, p<0.001 compared to non-diabetic (db/+) group. ##, p<0.005 compared to diabetic group supplemented with saline. B, Wound lipid peroxidation was measured using anti-hydroxynonenal (HNE) antibody and immunostaining. Data are mean ±SD (n = 4), **, p<0.01 compared to diabetic group supplemented with saline. C, Apoptotic cell count in wound tissue sections was measured using active caspase 3 immunohistochemical approach. Quantification (bar graphs) of caspase 3 positive area was performed using Image processing Tool kit. Data are shown as mean ± SD (n = 4).
(0.06 MB PDF)
