# Supplementary figures and images for: Macrophage Dysfunction Impairs Resolution of Inflammation in the Wounds of Diabetic Mice
Source: PLoS One. 2010 Mar 4;5(3):e9539. doi: 10.1371/journal.pone.0009539 (PMC2832020; doi:10.1371/journal.pone.0009539)

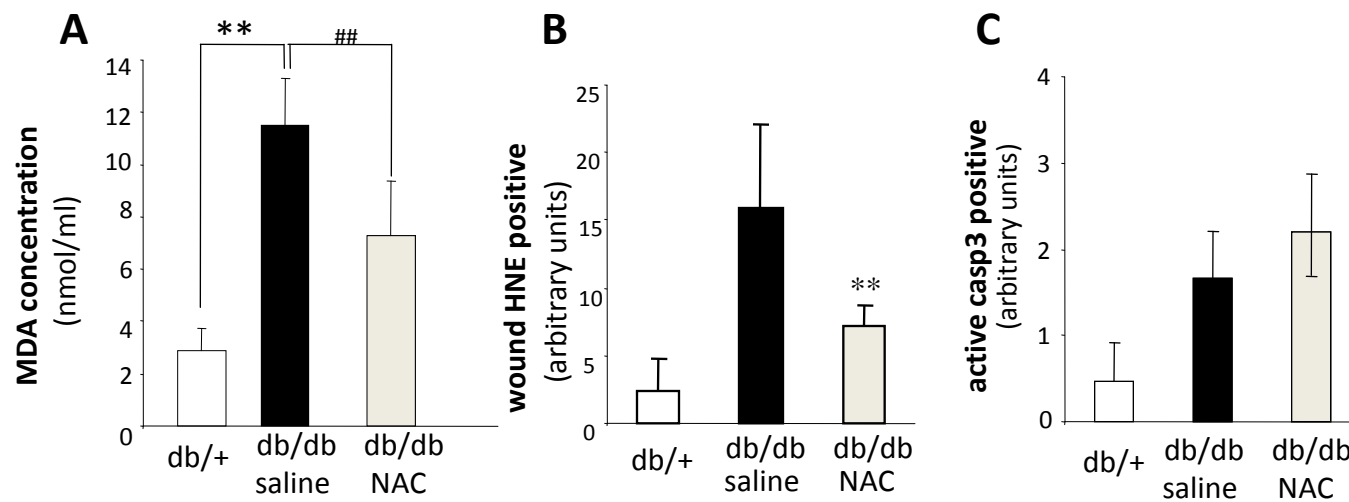

Figure S1

Supplement: Figure S1 — Antioxidant supplementation to diabetic mice attenuates oxidative stress but does not influence apoptotic cell count in wound tissue. Diabetic db/db mice were supplemented with N-acetyl-cysteine (NAC, 1 mg/g body weight, daily once) for three weeks. The control db/db group was supplemented with matched volume of saline. At the end of three weeks blood glucose, body weight and plasma MDA levels were measured. Two excisional (8 mm punch) wounds were placed on the back of mice. NAC supplementation continued throughout the healing period. A, Plasma lipid peroxidation (MDA levels) as a marker of oxidative stress was measured. Data are mean ± SD; n = 6. **, p<0.001 compared to non-diabetic (db/+) group. ##, p<0.005 compared to diabetic group supplemented with saline. B, Wound lipid peroxidation was measured using anti-hydroxynonenal (HNE) antibody and immunostaining. Data are mean ±SD (n = 4), **, p<0.01 compared to diabetic group supplemented with saline. C, Apoptotic cell count in wound tissue sections was measured using active caspase 3 immunohistochemical approach. Quantification (bar graphs) of caspase 3 positive area was performed using Image processing Tool kit. Data are shown as mean ± SD (n = 4). (0.06 MB PDF) [file pone.0009539.s001.pdf]
